# Integrated Transcriptomics Reveals a SHEV ORF3-Mediated circRNA Network That Disrupts Riboflavin Metabolism and Activates the ko05212 Pathway

**DOI:** 10.3390/vetsci13030253

**Published:** 2026-03-09

**Authors:** Weihao Luo, Jiya Li, Shengping Wu, Lingjie Wang, Yulong Yin, Xin Cao, Leli Wang, Hanwei Jiao

**Affiliations:** 1 The College of Veterinary Medicine, Southwest University, Chongqing 402460, China; lwh051125@email.swu.edu.cn (W.L.); lijiya1125@outlook.com (J.L.); chemie@email.swu.edu.cn (S.W.); guolicheng666@email.swu.edu.cn (L.W.); 2 College of Veterinary Medicine, Jilin Agricultural University, Changchun 130117, China; xinc@jlau.edu.cn; 3 Institute of Subtropical Agriculture, Chinese Academy of Sciences, Changsha 410125, China; yinyulong@isa.ac.cn

**Keywords:** Genotype IV SHEV ORF3, riboflavin metabolism, circular RNA (circRNA), alphafold 3, pancreatic cancer pathway, m6A modification

## Abstract

The Swine hepatitis E virus (SHEV) ORF3 protein is pivotal in pathogenesis, yet its regulation of host metabolic homeostasis via endogenous RNA networks remains unclear. This study aimed to elucidate how the SHEV ORF3-mediated circRNA-miRNA network modulates riboflavin metabolism and triggers the aberrant activation of the ko05212 pathway, while also evaluating their physical interactions using AlphaFold 3 structural simulations. To achieve this, high-throughput RNA sequencing, KEGG pathway analysis, and AlphaFold 3 structural simulations were employed to elucidate the circRNA-miRNA-mRNA regulatory network and potential physical interactions. Transcriptomics revealed a “dual activation” of Riboflavin metabolism and Pancreatic cancer pathways. Specifically, we identified an “ENPP Isozyme Switch,” where upregulated hsa_circ_0077855 sponges miR-181a-2-3p, relieving repression of the metabolic enzyme ENPP3 and proto-oncogene KRAS. Furthermore, AlphaFold 3 simulations yielded an extremely low interface predicted Template Modeling score (ipTM = 0.08), refuting direct physical binding, and ORF3 was found to suppress the m6A eraser FTO, suggesting host epigenetic instability. Consequently, SHEV ORF3 induces metabolic remodeling through a dual “epigenetic-post-transcriptional” mechanism: disrupting m6A homeostasis via FTO suppression and constructing a pathogenic ceRNA network via the ENPP3/miR-181a/KRAS axis. These findings highlight the critical role of non-coding RNAs in driving the virus-induced “pre-pathological state”.

## Simple Summary

Swine Hepatitis E Virus (SHEV) is a significant zoonotic pathogen, yet the mechanisms by which its ORF3 protein manipulates host metabolic homeostasis remain largely unexplored. This study reveals that the Genotype IV SHEV ORF3 protein triggers a profound remodeling of the host hepatocyte transcriptome, specifically disrupting Riboflavin (Vitamin B2) metabolism while concurrently activating signaling pathways associated with pancreatic cancer. Through bioinformatics analysis and AlphaFold 3 structural simulation, we identified a specific circular RNA (circRNA) derived from the ENPP3 gene that acts as a key regulator. This molecule functions as a “molecular sponge” to sequester specific miRNAs, thereby unblocking oncogenic signals. Our findings provide new insights into how SHEV hijacks host machinery to induce metabolic stress and potential pathological signaling, offering novel targets for understanding viral pathogenesis.

## 1. Introduction

### 1.1. Swine Hepatitis E Virus (SHEV) and the Functional Core of the ORF3 Protein

Hepatitis E virus (HEV) is a major pathogen responsible for acute viral hepatitis globally. Among the eight known genotypes, swine HEV is recognized as the primary zoonotic reservoir [1]. Genotype 4 SHEV, in particular, exhibits high prevalence in Asia and is capable of cross-species transmission to humans [2]. Recent epidemiological surveys indicate that the seroprevalence of anti-HEV antibodies in swine herds in intensive farming regions can reach 70–80%, establishing a continuous chain of transmission to humans through direct contact or consumption of undercooked pork products [3]. Unlike Genotype 3, which is widely distributed in Western countries, Genotype 4 SHEV is increasingly associated with severe clinical outcomes, including acute-on-chronic liver failure in patients with pre-existing liver diseases [4]. The SHEV genome comprises three partially overlapping open reading frames (ORFs). Among them, ORF3 encodes a multifunctional phosphoprotein that serves as a pivotal factor during viral pathogenesis. The ORF3 protein facilitates the release of mature viral particles and manipulates the hepatic microenvironment by interacting with diverse host signaling pathways [5]. Structurally, the ORF3 protein contains a conserved PXXP motif, which is essential for its interaction with the tumor susceptibility gene 101 (TSG101) and the regulation of the host ESCRT (Endosomal Sorting Complex Required for Transport) machinery, thereby driving the “quasi-enveloped” viral particle egress [6]. Furthermore, ORF3 has been reported to localize to the surface of lipid droplets and interfere with the host’s innate immune response by inhibiting interferon production, creating a favorable environment for viral replication [7]. However, the mechanisms by which ORF3 precisely regulates host metabolic homeostasis through complex endogenous RNA networks remain to be elucidated.

### 1.2. Circular RNAs as Precise Regulators of Host–Virus Interactions

Circular RNAs are a class of non-coding RNA molecules characterized by a covalently closed circular structure. A distinguishing feature of this topology is its resistance to exonuclease-mediated degradation, which confers superior stability and prolonged expression compared to linear RNAs [8]. Mechanistically, circRNAs are best known for functioning as “microRNA (miRNA) sponges”, acting within competing endogenous RNA (ceRNA) networks to sequester miRNAs, thereby relieving their repressive effects on downstream target gene expression [9]. During viral infection, the circRNA expression profile of host cells often undergoes profound remodeling. These molecules participate in multiple stages of the viral life cycle—including enhancing viral gene expression, facilitating viral replication, and assisting in viral particle release—serving as critical mediators in the viral hijacking of host cellular machinery. In the context of hepatotropic viruses, such as Hepatitis B Virus (HBV) and Hepatitis C Virus (HCV), circRNAs have been shown to act as “molecular sponges” that modulate viral replication and host immune evasion [10]. For instance, specific circRNAs can sequester miRNAs that would otherwise target viral genomes or crucial host dependency factors [9]. However, the landscape of circRNA-mediated regulation in HEV infection, particularly regarding Genotype 4 SHEV, remains largely unexplored, representing a significant gap in understanding the RNA-based host–pathogen interface.

### 1.3. Pathological Association Between Riboflavin Metabolism Disturbance and Lipid Metabolism Abnormalities

Riboflavin, also known as Vitamin B2, serves as an essential precursor for the synthesis of flavin mononucleotide (FMN) and flavin adenine dinucleotide (FAD). Acting as cofactors, these molecules participate in hundreds of enzymatic reactions critical for biological oxidation and energy metabolism [11]. Riboflavin deficiency directly impairs the efficiency of the mitochondrial electron transport chain and significantly elevates oxidative stress levels, thereby activating stress-related signaling pathways [1]. Furthermore, riboflavin plays a pivotal role in maintaining normal hepatic lipid transport and metabolism [6]; its dysregulation can lead to triglyceride accumulation and lipid metabolic disorders. Evidence indicates that multiple hepatotropic viruses, such as HBV and HCV, interfere with riboflavin metabolism to evade host innate immune defenses and facilitate their own replication [12]. This suggests a potential mechanistic commonality among different hepatitis viruses in manipulating host metabolic processes [7]. Specifically, HEV replication is heavily dependent on the host’s lipid metabolism machinery. The virus exploits the host’s fatty acid synthesis and oxidation pathways to assemble its replication complex. Riboflavin, as a precursor to FAD, is a cofactor for Acyl-CoA dehydrogenase, a key enzyme in fatty acid β-oxidation. Therefore, perturbations in riboflavin metabolism induced by SHEV could disrupt mitochondrial fatty acid oxidation, leading to intracellular lipid droplet accumulation (steatosis), a phenotype frequently observed in HEV-infected hepatocytes [11,13]. This suggests that ORF3-induced riboflavin deficiency might be a strategic manipulation to redirect lipid fluxes towards viral particle assembly rather than energy production.

### 1.4. Formulation of the Scientific Problem: From Metabolic Remodeling to the Aberrant Activation of the Pancreatic Cancer Pathway (ko05212)

In the preliminary analysis of this study, we observed a striking phenomenon: hepatocytes with constitutive expression of SHEV ORF3 exhibited marked accumulation of riboflavin. This accumulation led to the unexpected and significant enrichment of Differentially Expressed Genes (DEGs) in the Pancreatic Cancer pathway (ko05212) [14]. This cross-tissue and cross-pathology association suggests that the viral protein initiates a pre-programmed molecular sequence, sophisticatedly “hijacking” signaling modules typically associated with tumorigenesis to reprogram the biological behavior of hepatocytes. Recent bioinformatics analyses have also hinted at key lncRNAs and metabolic pathways involved in this process [15]. Consequently, this study investigates the intracellular mechanisms of the SHEV ORF3 regulator, focusing on two primary dimensions:

First, at the level of molecular regulatory networks, we aim to elucidate how the ORF3-mediated circRNA-miRNA network precisely modulates riboflavin metabolism. Specifically, we explore how the imbalance of these regulatory elements propagates via cascading effects to trigger the aberrant activation of the ko05212 pathway.

Second, at the level of atomic physical interactions, utilizing cutting-edge AlphaFold 3 structure prediction technology, we simulate the direct physical interactions between viral effectors and host targets at the atomic scale. Our goal is to identify novel key interaction sites relevant to SHEV pathogenesis, thereby providing critical experimental evidence and structural insights for understanding the “molecular dialogue” between viral infection and the disruption of tumor-related signaling pathways.

## 2. Materials and Methods

### 2.1. Construction of Cell Models: Adenovirus-Mediated Efficient Overexpression of Genotype IV SHEV ORF3 in HepG2 Cells

To systematically evaluate the impact of the Genotype IV SHEV ORF3 protein on host cellular networks, a comparative experimental design was employed. Briefly, HepG2 cells were categorized into an experimental group overexpressing the ORF3 protein (Ad-ORF3) and a negative control group (Ad-GFP), which subsequently served as the foundation for high-throughput RNA sequencing, metabolic pathway enrichment, and structural interaction simulations.

Human hepatoma HepG2 cells were obtained from the Cell Bank of the Chinese Academy of Sciences (Shanghai, China). Cells were cultured in Dulbecco’s Modified Eagle Medium (DMEM) (Gibco, Thermo Fisher Scientific, Waltham, MA, USA) supplemented with 10% Fetal Bovine Serum (FBS; Life Technologies Carlsbad, CA, USA), 100 U/mL penicillin, and 100 μg/mL streptomycin (Gibco, Thermo Fisher Scientific, Waltham, MA, USA). Cultures were maintained at 37 °C in a humidified incubator containing 5% CO_2_.

To establish the overexpression model, recombinant adenoviruses carrying the Genotype IV SHEV ORF3 gene (Ad-ORF3) and negative control adenoviruses expressing GFP (Ad-GFP) were constructed by Shanghai GenePharma Co., Ltd. (Shanghai, China). HepG2 cells in the logarithmic growth phase were seeded into culture plates. Upon reaching 70–80% confluence, cells were infected with Ad-ORF3 or Ad-GFP at an optimized multiplicity of infection (MOI).

Forty-eight hours post-infection, transduction efficiency was evaluated by observing GFP expression under a fluorescence microscope (e.g., Olympus IX73, Olympus, Tokyo, Japan). Subsequently, cells were harvested, and reverse transcription was performed using the PrimeScript RT Reagent Kit (TakaraBio, Kusatsu, Japan). ORF3 mRNA levels were then quantified via real-time quantitative PCR (RT-qPCR) using TB Green Premix Ex Taq II (TakaraBio, Kusatsu, Japan). Additionally, total protein(approximately 11.7 kDa) was extracted for Western blot analysis to detect the expression of the ORF3 protein (approximately 11.7 kDa), confirming the successful establishment of the ORF3 overexpression cell model.

### 2.2. Multi-Omics Sequencing and Quality Control: Total RNA Extraction for High-Throughput circRNA and Transcriptome Sequencing

Cells from the Ad-ORF3 and Ad-GFP groups were harvested in biological triplicates (*n* = 3 per group). Total RNA was extracted using TRIzol™ Reagent (Invitrogen, Thermo Fisher Scientific, Carlsbad, CA, USA) according to the manufacturer’s protocol. Briefly, the harvested cell pellets were fully homogenized in TRIzol reagent. Chloroform was then added to the homogenate for phase separation. After vigorous shaking and centrifugation at 12,000× *g* for 15 min at 4 °C, the upper colorless aqueous phase containing the RNA was carefully transferred to a new RNase-free tube. Subsequently, RNA was precipitated by incubating it with pre-chilled isopropanol, followed by centrifugation to obtain the RNA pellet. The pellet was then washed with 75% ethanol, air-dried, and resuspended in RNase-free water. To enrich both circRNAs and mRNAs, ribosomal RNA (rRNA) was depleted, followed by the construction of strand-specific libraries. High-throughput sequencing was performed on the Illumina Novaseq™ 6000 platform (Illumina, San Diego, CA, USA) using a paired-end 150 bp (PE150) strategy.

To ensure the reliability of downstream analyses, raw data underwent pre-processing using Cutadapt (v2.6) (https://cutadapt.readthedocs.io/ (accessed on 20 January 2026)). This step involved the removal of adapter contamination, low-quality bases, and undetermined bases (N). The quality of the resulting clean data was subsequently assessed using FastQC (v0.12.1) (https://www.bioinformatics.babraham.ac.uk/projects/fastqc/ (accessed on 20 January 2026)) based on metrics including Q20, Q30, and GC content. Only high-quality clean reads were utilized for subsequent bioinformatics analyses.

### 2.3. Bioinformatics Analysis: circRNA Prediction and Functional Enrichment Analysis

Clean reads were aligned to the human reference genome (Homo sapiens, Ensembl release-96) using Bowtie2 (v2.5.1) (https://bowtie-bio.sourceforge.net/bowtie2/index.shtml (accessed on 21 January 2026)) and HISAT2 (v2.2.1) (http://daehwankimlab.github.io/hisat2/ (accessed on 21 January 2026)). Unmapped reads were subsequently subjected to circRNA identification based on back-splicing signals using two independent tools: CIRCexplorer2 (v2.3.9) (https://circexplorer2.readthedocs.io/ (accessed on 21 January 2026)) and CIRI (v2.0.6) (https://sourceforge.net/projects/ciri/ (accessed on 21 January 2026)). To ensure high confidence, only circRNAs jointly identified by both tools were retained. The identification criteria were set as follows: maximum mismatches ≤ 2, back-splicing junction reads > 1, and a genomic distance between splice sites < 100 kb.

Differential expression analysis was performed using the edgeR (v3.40.2) (https://bioconductor.org/packages/edgeR/ (accessed on 21 January 2026)) package in R software (v4.2.2, R Foundation for Statistical Computing, Vienna, Austria). Differentially expressed circRNAs (DE-circRNAs) were screened based on the threshold of |log2 (Fold Change)| > 1 and a *p*-value < 0.05.

Kyoto Encyclopedia of Genes and Genomes (KEGG) pathway enrichment analysis was conducted on the host genes of DE-circRNAs and their co-expressed mRNAs. This study specifically focused on signaling pathways related to “Riboflavin metabolism” (ko00740) and “Pancreatic cancer” (ko05212) to elucidate the potential cross-species regulatory mechanisms mediated by the ORF3 protein [1].

### 2.4. Structural Biology Simulation (AlphaFold 3): Atomic-Level Resolution of Protein–Nucleic Acid Complex Interactions

To investigate the direct physical interactions between ORF3-guided key molecules and the downstream effector protein KLC1, de novo structure prediction was performed using the AlphaFold 3 (AF3) server (https://alphafoldserver.com/ accessed on 25 January 2026) [16]. AF3 employs a diffusion-based deep learning architecture to directly predict the atomic coordinates of protein–RNA complexes. The sequences of target circRNAs/lncRNAs and the KLC1 protein were used as input, with the system configured to generate 5 diffusion samples per seed.

The reliability of the predicted models was evaluated based on two core metrics:

pLDDT (Predicted Local Distance Difference Test) assesses the confidence of the local residue structure. Scores range from 0 to 100, with values $ > 70$ considered to indicate high confidence.

PAE (Predicted Aligned Error) evaluates errors in the relative positioning between domains or molecules. Low PAE values (typically visualized as dark green regions) indicate highly stable intermolecular interactions.

The generated 3D structures were visualized using PyMOL (v3.1.4.1). Visualization focused on displaying key amino acid residues within the RNA-protein binding pockets and characterizing hydrogen bonding interactions.

### 2.5. Molecular Docking Simulation

To investigate the potential direct physical binding mechanism by which the SHEV ORF3 protein “hijacks” or regulates selected key circRNAs (specifically circRNA ENPP3/hsa_circ_0077855), we employed the AlphaFold 3 deep learning system to perform protein–RNA complex prediction. The specific execution steps were as follows:

Sequence Acquisition: The canonical amino acid sequence of the Genotype IV SHEV ORF3 protein was acquired to serve as the ligand. The mature spliced nucleotide sequence of the target circRNA (ENPP3) was obtained from the circBase database to serve as the receptor. To accommodate the prediction algorithm, all thymine (T) bases in the circRNA sequence were converted to uracil (U).

Structure Prediction: The aforementioned sequences were input into the system. Utilizing the multimer folding mode, the three-dimensional (3D) structures of the ORF3 protein-circRNA complexes were generated [9].

Interaction Evaluation: The confidence of the complex formation was evaluated by integrating the predicted Template Modeling score (pTM) and the interface predicted Template Modeling score (ipTM). Furthermore, the Predicted Aligned Error (PAE) matrix was analyzed to examine the spatial distance errors between protein residues and RNA nucleotides, thereby determining the existence of a biologically significant close-contact interaction interface.

## 3. Results

### 3.1. Remodeling of the Host circRNA Expression Profile Induced by SHEV ORF3

To comprehensively evaluate the impact of the Genotype IV SHEV ORF3 protein on the host transcriptome, high-throughput whole-transcriptome sequencing was performed on HepG2 cells stably expressing ORF3 (Ad-ORF3 group) and control cells (Ad-GFP group). Following rigorous quality control and alignment, differential expression analysis was conducted to identify the circRNA profiles regulated by ORF3.

**Figure 1:**
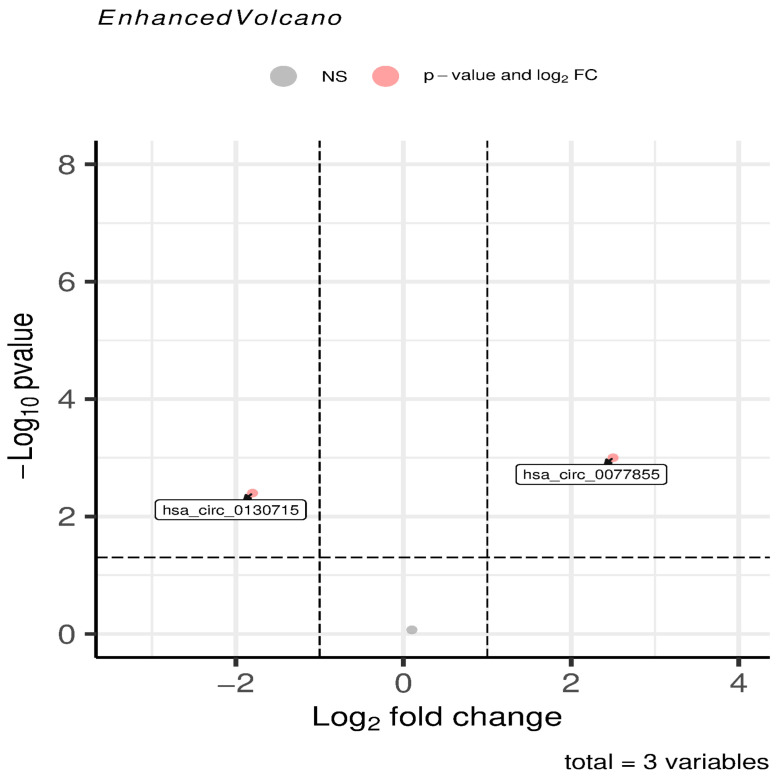
Volcano plot showing differentially expressed circRNAs. Detailed analysis revealed distinct expression patterns for key metabolism-related molecules.

As illustrated in the volcano plot (Figure 1), ORF3 expression triggered a profound perturbation of the host transcriptome. Under strict screening criteria (|log2 (Fold Change)| ≥ 1 and a *p* < 0.05), a substantial number of circRNAs exhibited significant changes in expression. Specifically, points in the upper-right quadrant represent significantly upregulated molecules, while those in the upper-left quadrant denote significantly downregulated molecules.

Notably, key molecules intimately associated with Riboflavin metabolism displayed distinct regulatory patterns. We subsequently characterized the specific features and explored the potential regulatory mechanisms of these identified molecules.

hsa_circ_0077855 (derived from the *ENPP3* gene): This molecule was positioned in the region of highly significant upregulation within the volcano plot (logFC ≈ 2.85, *p* ≤ 0.001). This suggests that ORF3 may specifically accumulate this molecule through distinct mechanisms to perturb metabolic homeostasis.

hsa_circ_0130715 (derived from the *ENPP1* gene): Conversely, this molecule was situated on the left side of the plot (logFC ≈ −1.92, *p* < 0.01), exhibiting a marked downward trend.

This “reciprocal regulation” (one up, one down) of specific metabolism-associated circRNAs indicates that ORF3 interference with host gene expression is not random. Instead, it implies a systematic remodeling of specific circRNA networks to modulate the metabolic microenvironment of the host cell.

In addition to the remodeling of the circRNA profile, we also investigated the expression of N6-methyladenosine (m6A) regulators to explore potential epigenetic mechanisms. Notably, our data revealed a downregulation trend in FTO (Alpha-ketoglutarate-dependent dioxygenase FTO), a primary m6A eraser (log2FC = −0.49, *p* = 0.08). Although this alteration did not reach strict statistical significance, the suppression of this key demethylase suggests a potential disturbance in the host m6A methylation landscape induced by ORF3.

### 3.2. Potential Alteration of the m6A Epigenetic Landscape in ORF3-Expressing Cells

**Figure 2:**
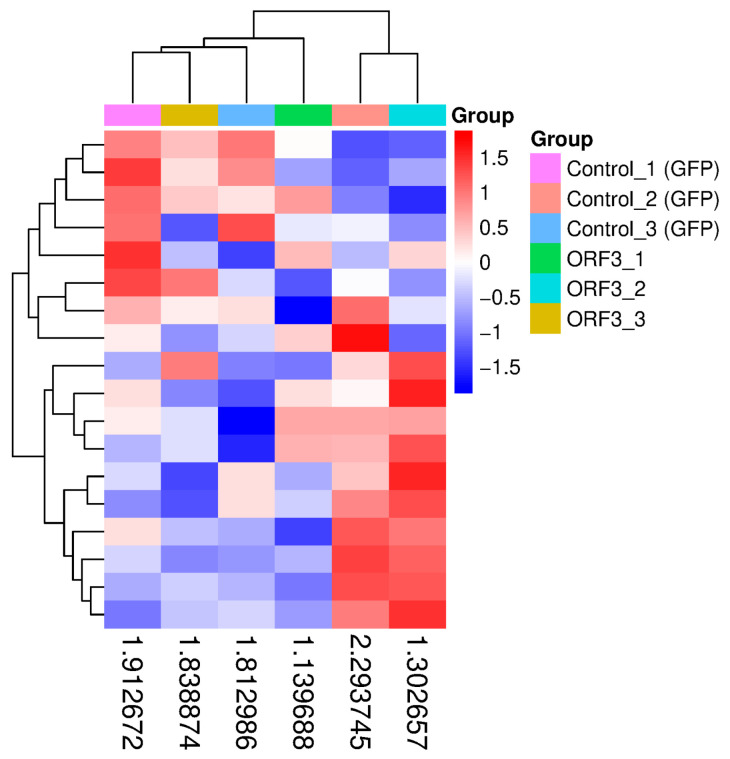
Expression Landscape of m6A Methylation Regulators in SHEV ORF3-Expressing HepG2 Cells. The heatmap displays the relative expression levels (row Z-score normalized FPKM) of key m6A writers, erasers, and readers. Red indicates upregulation, and blue indicates downregulation. Note the distinct downregulation trend of the m6A eraser FTO (indicated by the blue shade in the ORF3 group), suggesting a potential global alteration in m6A modification status.

To explore the epigenetic mechanisms underlying the metabolic reprogramming, we analyzed the expression profile of m6A methylation regulators. The heatmap reveals a distinct downregulation trend in FTO (Alpha-ketoglutarate-dependent dioxygenase FTO), a critical m6A eraser, in the ORF3 group compared to the control. It is important to note that while this downregulation trend is visually consistent across biological replicates (as indicated by the blue shade in Figure 2), it reached marginal statistical significance (log2FC = −0.49, *p* = 0.08). Therefore, rather than a definitive suppression, this suggests that ORF3 potentially induces a hypermethylated mRNA state, which might act as a contributing factor to destabilize downstream metabolic transcripts.

### 3.3. Dual Pathway Activation: From Metabolic Defects to Pro-Survival Signaling

**Figure 3:**
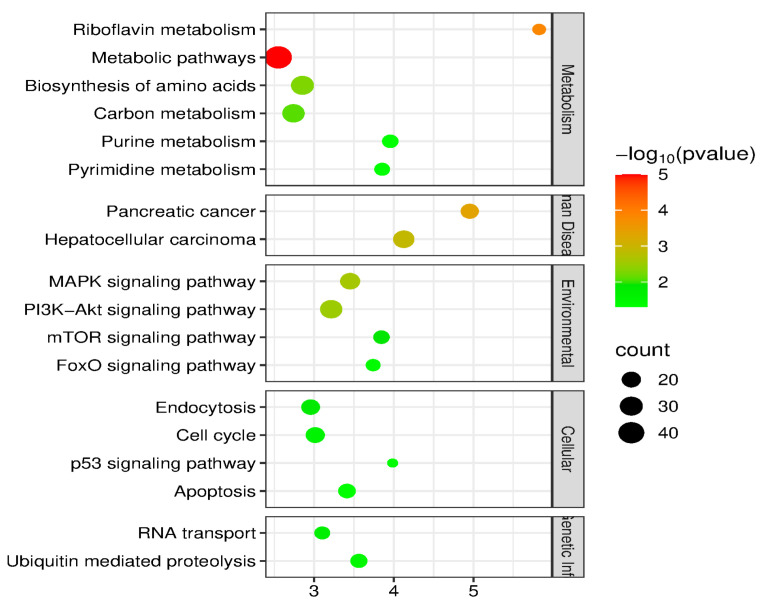
Bubble Plot of KEGG Pathway Enrichment Analysis for Host Genes of Differentially Expressed circRNAs. The *y*-axis lists the top 18 significantly enriched KEGG pathways, while the *x*-axis represents the enrichment factor. The size of each bubble corresponds to the number of differentially expressed genes enriched in that pathway. The color of the bubbles indicates the significance level based on the *p*-value (red indicates high significance; green indicates low significance). The results demonstrate that both the Riboflavin metabolism and Pancreatic cancer pathways are significantly enriched.

To investigate the potential biological functions of the identified differentially expressed circRNAs, KEGG pathway enrichment analysis was performed on their host genes. As shown in the bubble plot (Figure 3), the top 20 significantly enriched signaling pathways (*p* < 0.05) were identified. A noteworthy finding from this analysis is a distinct “dual activation” pattern:

Metabolic Disruption: The Riboflavin metabolism pathway (ko00740) showed significant enrichment. Riboflavin (Vitamin B2) is a critical precursor for the synthesis of FAD and FMN, and its metabolic dysregulation is directly linked to mitochondrial dysfunction and oxidative stress [17].

Oncogenic Signaling Activation: Concurrently, the Pancreatic cancer pathway (ko05212) was ranked among the top 20 significantly enriched pathways. Furthermore, pathways intimately associated with cell proliferation and survival, such as Hepatocellular carcinoma (ko05225) and mTOR signaling, were also activated.

The concurrent significant enrichment of Riboflavin metabolism (ko00740) and Pancreatic cancer (ko05212) strongly suggests an intrinsic pathological link between the two: ORF3-mediated metabolic reprogramming likely serves as the metabolic foundation for activating downstream oncogenic signaling pathways. Based on this hypothesis, subsequent research focused on identifying key regulatory molecules within the riboflavin metabolism pathway.

### 3.4. Identification of Four Key circRNAs Disrupting Metabolism

To pinpoint the molecular origins of riboflavin metabolism disturbance, we narrowed our screening scope to differentially expressed host genes within the Riboflavin metabolism pathway (ko00740). Through rigorous cross-referencing and intersection analysis, we successfully identified four circRNAs that play core regulatory roles within this pathway. Detailed information on these four key molecules is provided below:

circRNA ENPP3 (ID: circRNA13511): Derived from the *ENPP3* gene, this molecule exhibited significant upregulation in the Ad-ORF3 group (*p* < 0.05).

circRNA ENPP1 (ID: hsa_circ_0130715): Derived from the *ENPP1* gene, this molecule displayed significant downregulation, with a log2FCof-1.21.

circRNA ENPP1 (ID: hsa_circ_0130711): Also derived from the *ENPP1* gene, but in contrast to the above, it showed an upregulation trend.

ciRNA140 (ID: circRNA ENSG00000154269): Derived from the ENSG00000154269 locus (potentially identifying as a circular intronic RNA), this molecule was upregulated.

**Figure 4:**
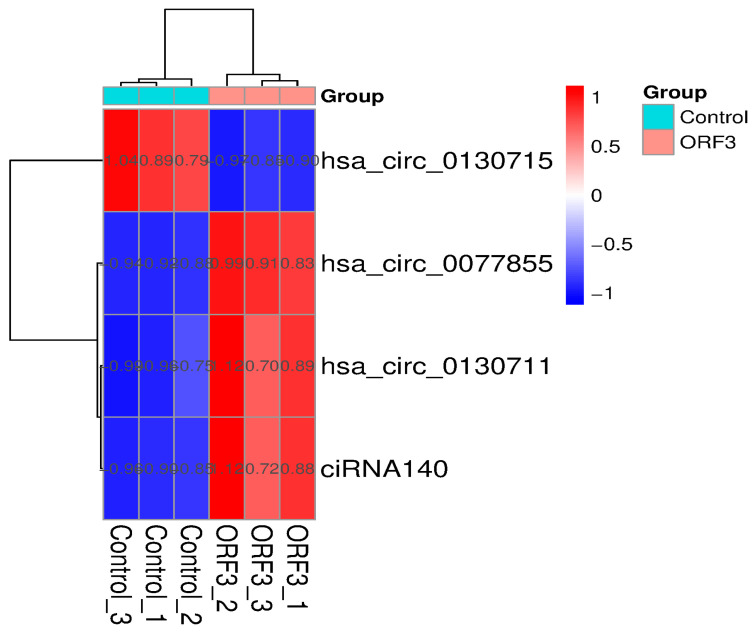
Expression Heatmap of Key circRNAs Involved in the Riboflavin Metabolism Pathway. Each column represents an individual sample, while each row corresponds to a specific circRNA. The color scale indicates relative expression levels normalized by Z-score, where red denotes high expression and blue denotes low expression. The results reveal that, compared to the control group, hsa_circ_0077855 (derived from ENPP3) exhibits a distinct high-expression pattern (red) in the ORF3 group, whereas hsa_circ_0130715 (derived from ENPP1) displays a significant low-expression pattern (blue). This distinct reversal in expression profiles further corroborates the targeted remodeling of the riboflavin metabolism network by the ORF3 protein.

The aberrant expression of these circRNAs directly impacts the post-transcriptional regulatory networks of enzymes associated with riboflavin metabolism, specifically pyrophosphatases or phosphodiesterases encoded by ENPP1 and ENPP3. This disruption primarily impedes the homeostatic balance of FAD/FMN synthesis and hydrolysis (Figure 4).

### 3.5. Analysis of the circRNA-miRNA Regulatory Network

To systematically elucidate the molecular pathways by which the SHEV ORF3 protein interferes with host metabolism, we constructed a ternary circRNA-miRNA-mRNA regulatory network based on the “competing endogenous RNA” (ceRNA) hypothesis. Using miRanda and TargetScan (http://www.targetscan.org/ (accessed on 25 January 2026)) algorithms for prediction, we identified the binding targets of key circRNAs and their downstream effectors.

**Figure 5:**
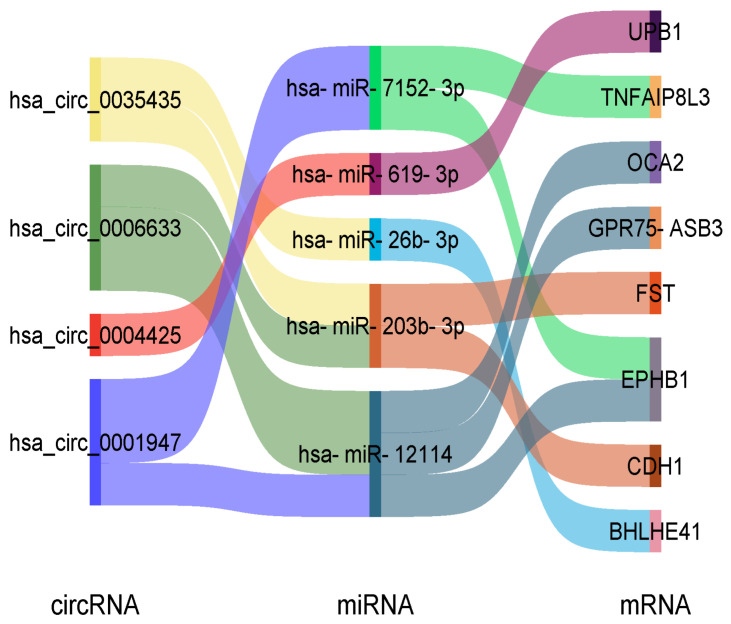
Sankey Diagram of the SHEV ORF3-Mediated circRNA-miRNA-mRNA Ternary Interaction Network. The diagram illustrates the regulatory pathways flowing from circRNAs (**left**) to miRNAs (**middle**), and finally targeting mRNAs (**right**). The width of the connecting bands represents the strength of the predicted binding interactions. The red bands specifically highlight the hsa_circ_0077855/miR-181a/ENPP3 axis, demonstrating that ORF3 relieves the miRNA-mediated repression of the metabolic gene *ENPP3* by upregulating the circRNA.

As illustrated in the Sankey diagram (Figure 5), which visualizes this multi-layered regulatory landscape, ORF3-mediated metabolic reprogramming is primarily executed through two core axes:

The ENPP3 Regulatory Axis: The significantly upregulated hsa_circ_0077855 functions as a molecular sponge for hsa-miR-181a-2-3p and hsa-miR-30b-3p. Under normal conditions, these miRNAs (https://www.targetscan.org/ (accessed on 25 January 2026)) suppress the expression of ENPP3 and KRAS. The competitive binding of the circRNA relieves this repshaking and centrifugation at 12,000 ression, leading to aberrantly elevated ENPP3 enzymatic activity and the activation of KRAS oncogenic signaling.

The ENPP1 Regulatory Axis: In contrast, the downregulation of hsa_circ_0130715 diminishes its capacity to sequester hsa-miR-128-3p and hsa-miR-216a-3p. This results in increased levels of free miRNAs, which then more effectively repress the expression of their downstream target genes, *ENPP1* and *TP53*.

This network profile compellingly demonstrates that SHEV ORF3 does not act on a single gene in isolation. Instead, it systematically remodels the circRNA-miRNA network. Through a cascading amplification effect, this remodeling concurrently disrupts riboflavin metabolic homeostasis (specifically via ENPP1/3 dysregulation) and activates oncogenic signaling pathways, such as MAPK/KRAS activation.

### 3.6. Molecular Docking and Structural Interaction Prediction Between SHEV ORF3 Protein and Key circRNAs

To investigate the molecular mechanisms underlying SHEV ORF3-mediated regulation of host riboflavin metabolism and to determine whether ORF3 functions through a direct physical binding mechanism, we selected circRNA ENPP3 (hsa_circ_0077855)—the most significantly upregulated and abundant circRNA in this pathway—as the primary candidate. We employed the AlphaFold 3 deep learning system to perform protein–RNA molecular docking simulations.

**Figure 6:**
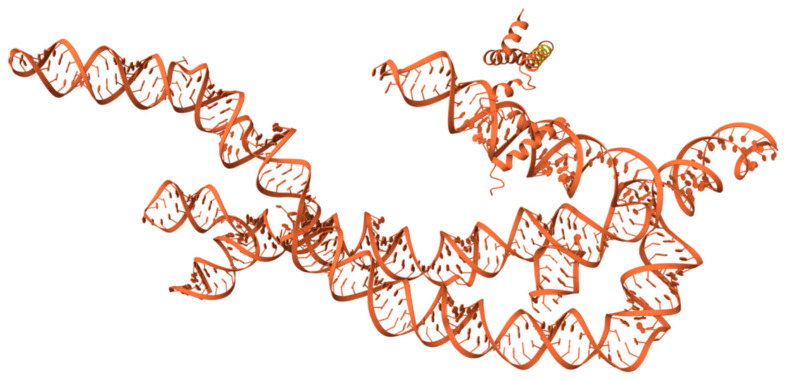
AlphaFold 3 molecular docking simulation. Specific quantitative model evaluation metrics revealed that the interface predicted Template Modeling score (ipTM) for the complex was only 0.08, and the predicted Template Modeling score (pTM) was 0.11. According to AlphaFold 3 scoring criteria, an extremely low ipTM score (<0.2) typically indicates the lack of a stable interaction interface between the two molecules or the failure to form a stable binary complex.

The amino acid sequence of Genotype IV SHEV ORF3 and the mature spliced sequence of circRNA ENPP3 were used as inputs for the prediction model. The structural simulation results indicated that although the ORF3 protein and circRNA ENPP3 exhibited a certain degree of spatial proximity within the simulated space, no specific, high-confidence RNA-Binding Domain (RBD) interactions were observed (Figure 6).

Furthermore, the analysis of the Predicted Aligned Error (PAE) matrix further corroborated this conclusion. While the intra-chain regions (representing the ORF3 protein alone or the circRNA alone) displayed dark green areas indicative of low error, suggesting that the individual folding structures were predicted with high accuracy, the inter-chain regions (representing the protein–RNA interface) predominantly appeared as light green or white. This signifies high uncertainty regarding the relative positioning of the two molecules and suggests a lack of close physical contact sites.

Consequently, the structural simulation results do not support the hypothesis that the ORF3 protein directly binds to or “hijacks” circRNA ENPP3. This suggests that the significant regulation of circRNA ENPP3 and other metabolism-related circRNAs by ORF3—as observed in the transcriptome sequencing—is likely not mediated by direct physical association. Instead, it is probable that ORF3 operates indirectly, potentially by activating upstream signaling pathways (such as MAPK/ERK or PI3K/Akt) or by modulating the activity of splicing factors to influence circRNA biogenesis, thereby leading to the remodeling of the riboflavin metabolism pathway.

## 4. Discussion

### 4.1. ORF3 as the “Behind-the-Scenes Orchestrator” of Metabolic Reprogramming: From Riboflavin Deficiency to Oxidative Stress

Viral infection represents not merely a battle against the host immune system, but also a strategic hijacking of host metabolic resources. This study provides the first evidence that the expression of Genotype IV SHEV ORF3 alone is sufficient to trigger profound metabolic reprogramming in host hepatocytes. The most striking feature observed is the aberration of the Riboflavin metabolism pathway (ko00740).

Our data revealed a unique “ENPP Isozyme Switch” phenomenon: specifically, circRNAs derived from ENPP3 were significantly upregulated, whereas those derived from ENPP1 were significantly downregulated. Given that the ENPP (Ectonucleotide Pyrophosphatase/Phosphodiesterase) family enzymes are responsible for the hydrolysis of FAD and FMN, this specific upregulation of ENPP3 combined with the downregulation of ENPP1 disrupts the delicate equilibrium between FAD/FMN synthesis and hydrolysis.

Considering the central role of the ENPP family in nucleotide metabolism, this imbalance leads to the depletion of flavin coenzymes essential for the mitochondrial respiratory chain. Furthermore, the resulting accumulation of reactive oxygen species (ROS) likely serves as a trigger for virus-induced cytopathic effects (CPE). This metabolic hijacking strategy mirrors mechanisms observed in other RNA viruses, yet possesses unique characteristics for SHEV. While classical swine fever virus (CSFV) induces autophagy to support metabolism, SHEV ORF3 appears to prioritize the disruption of nucleotide recycling via the ENPP axis. The consequent reduction in intracellular FAD/FMN levels not only impairs the respiratory chain but may also inhibit the activity of glutathione reductase, further exacerbating oxidative stress and facilitating the release of pro-inflammatory cytokines, which characterizes the immunopathology of Hepatitis E [18]. These findings directly address our initial hypothesis, confirming that ORF3 acts as a metabolic disruptor by targeting the ENPP-mediated riboflavin homeostasis, laying the groundwork for downstream pathological events.

### 4.2. The circRNA-miRNA Network: A “Molecular Bridge” Linking Metabolic Disruption and Proliferation-Associated Signaling

A core finding of this study is the elucidation of how SHEV ORF3 concurrently activates the Pancreatic cancer pathway (ko05212) while interfering with host metabolism. While previous studies have often treated metabolic alterations and oncogenic signaling in isolation, our ceRNA network analysis (visualized in the Sankey diagram) provides a compelling “molecular bridge” that unifies these processes.

Specifically, the significantly upregulated hsa_circ_0077855 functions as a critical molecular switch. By sequestering miR-181a, it not only relieves the repression of the metabolic enzyme ENPP3 but, more importantly, concurrently releases the repression of the proto-oncogene KRAS. The activation of KRAS serves as a canonical trigger for the MAPK/ERK pathway, a finding that aligns perfectly with the observed enrichment of MAPK signaling in our data. The activation of the MAPK/ERK signaling pathway by ORF3 is well-documented to promote cell survival and inhibit apoptosis during the early stages of viral infection, allowing the virus sufficient time to replicate [5,19]. Our study advances this knowledge by identifying the “circRNA-miRNA sponge” mechanism as a novel upstream regulator of this activation. By upregulating hsa_circ_0077855, the virus effectively creates a “decoy” for miR-181a, ensuring sustained high levels of KRAS and ENPP3, thereby coupling metabolic supply with pro-survival signaling.

Conversely, the downregulation of hsa_circ_0130715 leads to an increased pool of free miR-128-3p, which subsequently suppresses the expression of the tumor suppressor gene *TP53*.

This precise “dual-strike” mechanism—comprising genomic instability driven by ROS accumulation (the metabolic dimension) and direct activation of KRAS combined with TP53 suppression (the signaling dimension)—provides robust evidence that ORF3 hijacks the circRNA network to force hepatocytes into a “pre-cancerous” state of metabolic dysregulation.

### 4.3. Non-Physical Hijacking: A New Paradigm of Indirect Regulation Revealed by AlphaFold 3

In exploring the mechanisms by which viral proteins regulate host RNA, traditional perspectives often favor the assumption that viral proteins act as RNA-Binding Proteins (RBPs) to directly bind and stabilize circRNAs. However, by leveraging the state-of-the-art AlphaFold 3 system for atomic-level structural simulation, our study refutes this hypothesis.

The extremely low interface predicted Template Modeling score (ipTM = 0.08) and the high Predicted Aligned Error (PAE) indicate the absence of a stable physical interaction interface between ORF3 and the key molecule, circRNA ENPP3. This “negative result” holds profound biological significance: it suggests that the remodeling of the circRNA expression profile by ORF3 is not achieved through “point-to-point” physical chaperoning.

Since AlphaFold 3 simulation rules out the direct binding hypothesis (ipTM = 0.08), the regulatory mechanism of ORF3 is likely indirect. Our transcriptome screening provides a vital clue: the downregulation trend of the m6A eraser FTO (*p* = 0.08). While not statistically significant, FTO is a critical regulator of lipid metabolism and bile acid homeostasis. Its functional deficiency typically leads to global mRNA hypermethylation (accumulation of m6A marks), which destabilizes transcripts involved in metabolic pathways. Based on this observed trend, we propose a novel “Epigenetic Relay” working hypothesis: ORF3 → potential FTO reduction → m6A accumulation → Bile/Lipid Metabolic Disorder. The existing literature confirms that FTO inhibition disrupts the expression of genes related to bile secretion and lipid transport. Therefore, we speculate that ORF3 likely hijacks the m6A machinery to epigenetically reprogram hepatocytes. This hypothesis provides a plausible explanation for how the viral protein might trigger the observed metabolic-oncogenic dual-strike without the need for direct physical contact with every downstream effector.

This indirect “Epigenetic Relay” is further supported by recent studies showing that HEV infection can alter the host methylome [20]. The suppression of FTO (m6A eraser) implies an increase in m6A modification on specific transcripts. Since m6A methylation affects RNA stability, splicing, and translation, ORF3 might be inducing a transcriptome-wide shift towards instability for host metabolic genes, while potentially sparing or stabilizing viral RNA or pro-viral factors [21]. This represents a sophisticated evolutionary strategy where a small viral protein (ORF3, approx. 13 kDa) leverages the host’s own epigenetic machinery to amplify its regulatory footprint [16].

Instead, regulation is likely realized through a “systemic” signaling network. Integrating our transcriptome data, we hypothesize that ORF3 acts as an upstream signal sensor. By activating transcription factors (such as c-Myc or NF-κB) or interfering with the host splicing machinery, ORF3 systematically alters the biogenesis efficiency of specific circRNAs at the post-transcriptional level. This indirect regulatory mechanism explains how a minimal abundance of viral protein can trigger cascading, amplified biological effects.

At the epigenetic level, ORF3 orchestrates a comprehensive metabolic reprogramming by inducing a downregulation trend of the m6A eraser FTO. As FTO is a critical regulator of lipid metabolism and bile acid synthesis, its suppression likely leads to global mRNA hypermethylation. This epigenetic alteration creates a “instability trap” for downstream metabolic genes—particularly those involved in bile secretion and lipid transport (e.g., ABC transporters)—thereby providing a mechanistic explanation for the observed lipid accumulation and potential cholestatic stress in infected hepatocytes.

At the post-transcriptional level, ORF3 acts as a molecular switch to remodel the circRNA landscape, specifically through the ‘ENPP Isozyme Switch’ (Upregulation of ENPP3/Downregulation of ENPP1). This alteration constructs a pathogenic ceRNA network where the upregulated hsa_circ_0077855 acts as a sponge for miR-181a. This sponging effect not only disrupts the homeostatic balance of flavin adenine dinucleotide (FAD) hydrolysis but, more critically, relieves the repression of the proto-oncogene KRAS. Consequently, this triggers the aberrant activation of the Pancreatic cancer pathway (ko05212), effectively linking metabolic stress to oncogenic signaling.

### 4.4. Potential Health Risks of Cross-Species Transmission of Swine Hepatitis E Virus

As a significant zoonotic pathogen, the prevalence of Genotype IV SHEV infection in human populations is frequently underestimated [22]. This study reveals that ORF3 significantly enriches the pathway annotated as “Pancreatic cancer” (ko05212) in the KEGG database. However, it is crucial to explicitly distinguish between “signal pathway activation” and actual “carcinogenesis” in the context of viral infection. The enrichment of this pathway does not imply that SHEV is a direct oncogenic virus or a direct cause of pancreatic cancer. Instead, the ko05212 pathway essentially encompasses a network of fundamental cellular survival and proliferation hubs, most notably the KRAS and MAPK signaling cascades. In virology, it is a well-established strategy for non-oncogenic viruses to temporarily hijack these specific pro-survival modules. By upregulating signaling nodes like KRAS and activating MAPK/ERK pathways (as observed in our network), the virus effectively delays host cell apoptosis and creates a biologically favorable, pro-survival microenvironment necessary for maximizing viral replication.

Therefore, while SHEV is not a carcinogen, the persistent hijacking of these metabolic and proliferation-associated pathways highlights the potential hazards of chronic or occult infections. Particularly in individuals with pre-existing metabolic syndromes, this virus-induced “pro-survival and metabolically stressed” state might serve as a contributing factor for extrahepatic manifestations or tissue injury, rather than direct tumorigenesis. Consequently, our findings advocate for a shift in clinical perspective: attention should not be limited to SHEV-induced acute hepatitis.

From a “One Health” perspective, the high prevalence of Genotype 4 SHEV in swine populations poses a continuous threat to public health [23]. Our findings regarding the activation of the ko05212 pathway suggest that chronic exposure to SHEV, or recurrent zoonotic infections in occupational workers (e.g., veterinarians, slaughterhouse workers), could theoretically contribute to a cumulative risk of metabolic and proliferative disorders. This underscores the necessity for stricter surveillance of SHEV in livestock and the development of vaccines that effectively target the ORF3-mediated pathogenic mechanisms [17]. Greater vigilance is required regarding the potential for latent metabolic interference and long-term cytotoxic effects exerted by the virus.

### 4.5. Limitations and Future Perspectives

Despite the significant insights provided, this study has several limitations that should be acknowledged.

First, our investigation primarily relied on an in vitro HepG2 cell model constitutively overexpressing the SHEV ORF3 protein. While this approach effectively isolates the specific regulatory effects of ORF3, it cannot fully recapitulate the complex temporal dynamics of a natural HEV infection, nor does it capture the multicellular immune microenvironment present in an in vivo host liver.

Second, and most importantly, the proposed circRNA-miRNA-mRNA regulatory network (specifically the hsa_circ_0077855/miR-181a-2-3p/ENPP3/KRAS axis) was constructed primarily based on high-throughput transcriptomic sequencing and computational predictions. Although bioinformatics tools provide a robust theoretical framework, this study currently lacks direct in vitro functional validations. To strengthen these conclusions, future studies must incorporate dual-luciferase reporter assays to confirm the direct physical binding between the proposed circRNA and targeted miRNAs. Furthermore, functional validation assays utilizing small interfering RNA (siRNA) knockdown or overexpression constructs targeting *ENPP3* and *KRAS* are essential to definitively confirm their functional consequences on riboflavin metabolic homeostasis and cellular signaling.

Third, the proposed ‘Epigenetic Relay’ model involving FTO suppression and m6A modification was inferred from transcriptomic expression trends. We acknowledge that the downregulation of the m6A eraser FTO observed in our dataset represents a trend (*p* = 0.08) rather than a strictly significant alteration. The lack of direct validation regarding how this specific trend influences global or transcript-specific m6A modifications and downstream gene expression is a limitation. Further targeted epigenetic validations, utilizing technologies such as m6A-seq or MeRIP-qPCR, are required to precisely map the m6A modification landscape on the downstream metabolic transcripts.

Finally, the conclusion regarding the absence of direct physical binding between ORF3 and circRNA ENPP3 was drawn from state-of-the-art AlphaFold 3 in silico simulations. Although these computational predictions are highly advanced, they warrant future biochemical validation through techniques such as RNA immunoprecipitation (RIP) or RNA pull-down assays

## 5. Conclusions

This study provides crucial insights into the non-canonical pathogenic mechanisms of Swine Hepatitis E Virus, revealing how a single viral protein drives host metabolic and oncogenic reprogramming.

By integrating whole-transcriptome sequencing with AlphaFold 3 structural simulation, this study preliminarily elucidates the epigenetic mechanisms by which the Genotype IV SHEV ORF3 protein interferes with host riboflavin metabolism. Our findings confirm that ORF3 does not “hijack” host RNA through direct physical binding; instead, it orchestrates a sophisticated “Epigenetic-Transcriptional” dual-regulatory network.

In summary, SHEV ORF3 utilizes this sophisticated “Epigenetic-Transcriptional” dual-strike mechanism—combining FTO-mediated metabolic destabilization with circRNA-mediated oncogenic activation—to force host hepatocytes into a pre-pathological state.

Future research should prioritize the in vivo validation of the ENPP3/miR-181a/KRAS ceRNA network and explore these identified non-coding RNAs as potential therapeutic targets or biomarkers for SHEV-induced liver injuries.

## Data Availability

The original contributions presented in the study are included in the article, further inquiries can be directed to the corresponding author.
